# Systematic review and network meta-analysis of treatment strategies for asymptomatic carotid disease

**DOI:** 10.1038/s41598-018-22356-z

**Published:** 2018-03-13

**Authors:** Mohamed Barkat, Iain Roy, Stavros A. Antoniou, Francesco Torella, George A. Antoniou

**Affiliations:** 1 Liverpool Vascular and Endovascular Service, Royal Liverpool University Hospital, Liverpool, UK; 2 Institute of Ageing and Chronic disease, University of Liverpool, Liverpool, UK; 3 Department of Surgery, University Hospital of Heraklion, University of Crete, Heraklion, Greece; 4 School of Physical Sciences, University of Liverpool, Liverpool, UK; 5 Department of Vascular and Endovascular Surgery, The Royal Oldham Hospital, Pennine Acute Hospitals NHS Trust, Manchester, UK

## Abstract

We aim to perform a systematic review and meta-analysis to investigate outcomes of treatment strategies for asymptomatic carotid disease. We searched electronic bibliographic sources (MEDLINE, EMBASE, CINAHL and CENTRAL) to identify randomised controlled trials (RCT) reporting comparative outcomes of carotid endarterectomy (CEA), carotid stenting (CAS) and best medical therapy (BMT) in asymptomatic carotid disease. We performed pairwise meta-analysis applying random or fixed-effects models and reported the results as the odds ratio (OR) or risk difference (RD) and 95% confidence interval (CI). We also performed a network meta-analysis and obtained a hierarchy of the competing interventions using rankograms and the surface under the cumulative ranking curve and mean ranks. Stroke and death within 30 days and during follow up were the primary outcome endpoints. Eleven RCTs were identified reporting a total of 8,954 patients. Compared to BMT, CEA reduces the odds of long-term mortality (OR 0.70, 95% CI 0.43, 1.12) and ipsilateral stroke (OR 0.59 95% CI 0.50, 0.71). Network meta-analyses league table demonstrated that BMT is superior to CEA and CAS in terms of perioperative stroke risk and mortality. CEA is the preferred method to reduce the long-term risk of ipsilateral stroke and mortality for patients with asymptomatic carotid disease.

## Introduction

Stroke is the second leading cause of disability in Europe after ischaemic heart disease and the sixth leading cause worldwide^1^. In Europe, the annual cost of stroke is an estimated €27 billion: €18.5 billion for direct costs and €8.5 billion for indirect costs^2^.

Carotid atherosclerosis is a significant cause of ischemic stroke and transient ischemic attack (TIA)^3,4^. The optimal treatment for patients with asymptomatic carotid disease remains controversial with no clear consensus to recommend the best therapy for them. Carotid endarterectomy (CEA) has been suggested to be superior in preventing stroke compared to medical therapy alone in asymptomatic patients with >70% stenosis^5^. Carotid stenting (CAS) has emerged as a therapeutic alternative to endarterectomy for the treatment of severe carotid stenosis. The results of randomized trials comparing stenting and endarterectomy have been conflicting^6,7^.

In 2011, the American Heart Association (AHA) published its updated guidelines on the role of CEA and CAS in asymptomatic patients^8^. Its recommendations were based on two landmarks randomised clinical trials published in 1995 and 2004^9,10^. However, medical therapy in these trials was not comparable with current standards. Medical therapy for stroke prevention has improved since these original trials, with more widespread use of statins, more active lowering of blood pressure and more effective antiplatelet regimes. More recent data from the Oxford Vascular Study (OXVASC) demonstrate a stroke risk of only 0.3% per year attributable to ipsilateral asymptomatic carotid stenosis treated with best medical therapy (BMT) alone^11^.

In view of the improving medical therapy for stroke prevention with more widespread use of statins, more active lowering of blood pressure and more effective antiplatelet regimes, there is currently uncertainty as to whether carotid intervention (endarterectomy or stenting) provides clinical benefits and superior outcomes over optimised medical therapy in patient cohorts with asymptomatic carotid disease. Our systematic review investigated outcomes of treatment strategies for asymptomatic carotid disease applying network meta-analytic techniques.

## Results

### Literature search results

**Figure 1:**
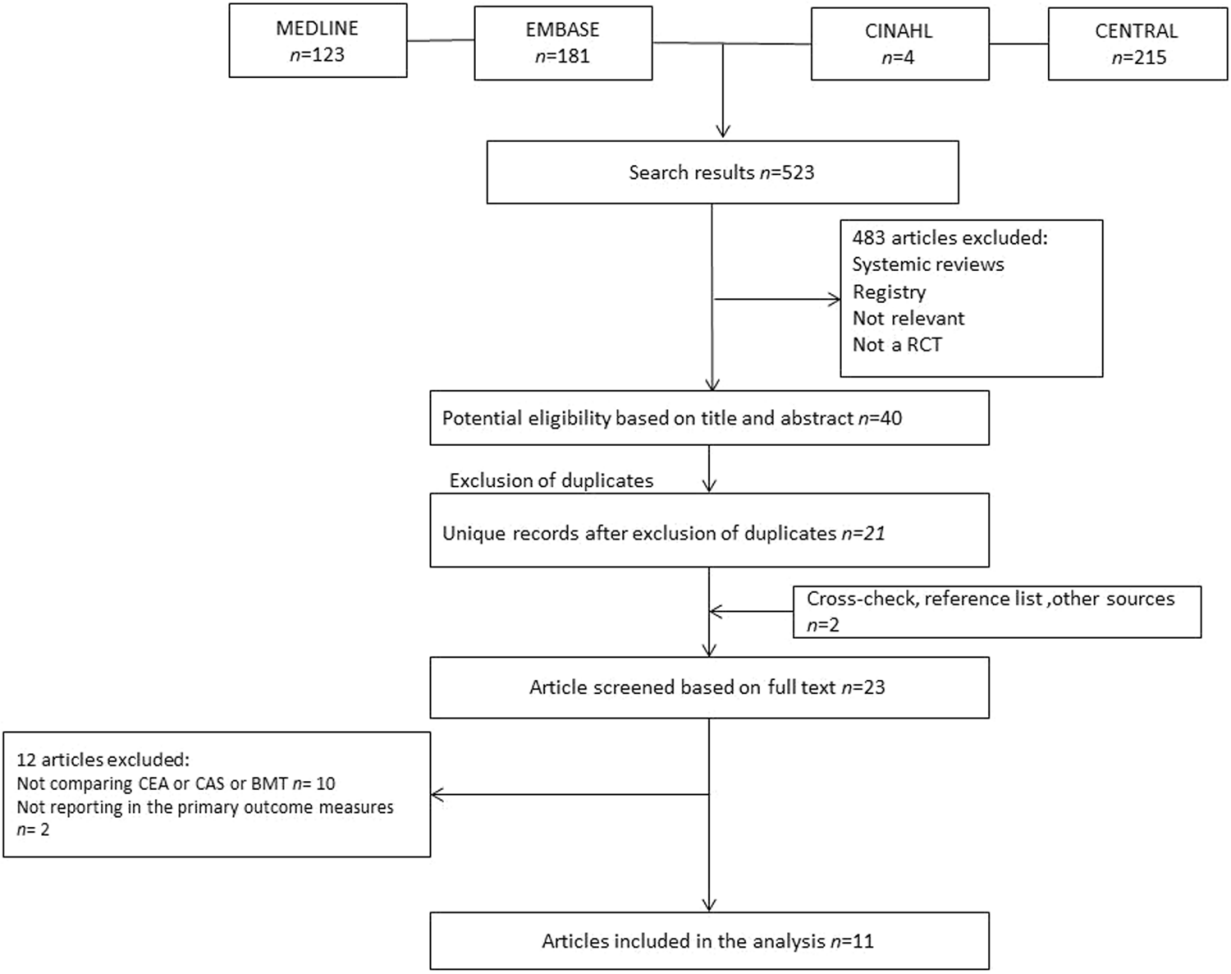
Study flow diagram. BMT, best medical therapy; CAS, carotid stenting; CEA, carotid endarterectomy; RCT, randomised controlled trial.

The initial literature search identified a total of 523 records. Two additional relevant records were identified through manual search of the references lists. Out of the 525 articles 23 articles were relevant to this study and the full-texts were assessed for eligibility criteria. Eleven articles^9,10,12–20^ met the inclusion criteria and were incorporated in the meta-analysis. Figure 1 summarizes the results of the literature search.

### Description of included studies

The characteristics of the selected studies and the specific study population baseline demographics and clinical characteristics are summarised in Tables 1 and 2, respectively.

**Table 1:** Study characteristics.

RCT	Recruitment period	Total number of patients	Patient groups	Antiplatelet therapy	Inclusion criteria	Primary endpoint	Follow up
**SPACE-2** ^12^ 2016 36 centres Austria, Germany, Switzerland	2009–2014	513	CEA: 203 CAS: 197 BMT: 113	CEA: Aspirin CAS: Aspirin + clopidogrel BMT: Aspirin	Patients aged 50–85 years with a 70–99% ICA stenosis based on ultrasound without stroke/TIA symptoms within the preceding 180 days	Primary safety endpoint: combined rate of death/any stroke within 30 days after CEA or CAS. The primary efficacy endpoint: cumulative rate of death/any stroke within 30 days plus the rate of ipsilateral ischemic stroke within 5 years of follow up	30 days 5 years
**ACT 1** ^13^ 2016 65 centres US	2005–2013	1453	CEA: 364 CAS:1089	CEA: Aspirin CAS: Aspirin + clopidogrel	Patients aged 79 years or younger with 70–99% ICA stenosis without symptoms during the previous 180 days. In the absence of substantial (>60%) contralateral carotid stenosis	Primary end point: 30-day incidence of stroke (Major or minor), death or MI. Or an ipsilateral stroke within 1 year.	5 years
**Mannheim Trial** ^14^ 2016 Haifa-Israel	No records	136	CEA: 68 CAS:68	CEA: Aspirin CAS: Aspirin + clopidogrel	Asymptomatic (for 6 months) patient with severe carotid atherosclerosis with >70% ICA stenosis	Primary end point was the 30-day incidence of stroke, death, MI and TIA	5 years
**AMTEC trial** ^15^ 2015 3 centres Russia	2009–2014	55	CEA: 31 BMT: 24	CEA: Aspirin BMT: Aspirin	Asymptomatic patient aged <80, with severe carotid atherosclerosis with 70–79% ICA stenosis, no stroke/TIA in last 6 months	Nonfatal ipsilateral stroke and death from any cause during a follow up period of 5 years.	3.3 years
**CREST** ^16^ 2011 108 centres US 9 centres Canada	2000–2008	1181	CEA: 587 CAS: 594	CEA: Aspirin CAS: Aspirin + clopidogrel or ticlopidine	ICA stenosis of ≥60% on angiography, ≥70% on ultrasound, or ≥80% on CTA/MRA if the stenosis on ultrasonography was 50 to 69%	The primary end point: any stroke, myocardial infarction, or death during the periprocedural period or ipsilateral stroke within 4 years	30 days 6 monthly for 4 years
**ACST-1** ^10^ 2010 126 centres 30 countries	1993–2003	3120	CEA:1560 BMT:1560	All patients Aspirin or Clopidogrel	Asymptomatic patient (last 6 months) with carotid stenosis of ≥60%	The primary endpoint: perioperative mortality and morbidity (death or stroke within 30 days) and non-perioperative stroke.	9 years
**Kentucky trial** ^17^ 2004 Kentucky US	1998–2002	85	CEA: 42 CAS: 43	All patients on Aspirin and Clopidogrel	Asymptomatic patient with carotid stenosis of more than 80%	The primary endpoint: perioperative mortality and morbidity (death or stroke within 30 days)	30 days 4 years 10 years
**SAPPHIRE trial** ^18^ 2004 29 centres US	2000–2002	237	CEA: 120 CAS: 117	CEA: Aspirin CAS: Aspirin + clopidogrel	Asymptomatic patients with at least 80% ICA stenosis on duplex ultrasonography and at least one coexisting condition that potentially increased the risk posed by carotid endarterectomy	The primary end point: the cumulative incidence of death, stroke, or MI within 30 days after the procedure or death or ipsilateral stroke between 31 days and 1 year.	30 days 1 year 3 years
**ACAS trial** ^9^ 1995 39 centres US/Canada	1987–1993	1659	CEA: 825 BMT: 834	CEA: Aspirin BMT: Aspirin	Patients aged 40–79 with asymptomatic ICA stenosis of ≥60%	The primary endpoints: TIA, all death/stroke within 30 days after CEA or 42 days in BMT group (to reflect 12 day delay to surgery). Efficacy end point: ipsilateral stroke in 5-year period	2.7 years
**Veterans Affairs trail** ^19^ 1993 11 centres US	1983–1987	444	CEA: 211 BMT: 233	CEA: Aspirin BMT: Aspirin	Patients with asymptomatic ICA stenosis of ≥50%)	The primary end point: cumulative incidence of TIA, death, stroke within 30 days after the procedure or death or stroke in 4 years	3.9 years
**MACE trial** ^20^ 1992 Mayo clinic US	Over 30 months	71	CEA: 36 BMT: 35	CEA: None BMT: Aspirin	Patients with asymptomatic carotid stenosis (≥50% linear stenosis or 75% cross sectional area stenosis)	The primary end point of the trial was the cumulative incidence of TIA, any stroke and death.	1.9 years

BMT, best medical therapy; CAS, carotid stenting; CEA, carotid endarterectomy; CTA, computed tomographic angiography; ICA, internal carotid artery; MI, myocardial infarction; MRA, magnetic resonance angiography; RCT, randomised controlled trial; TIA, transient ischemic attack.

**Table 2:** Patient demographic and clinical characteristics.

RCT	Age (mean+/− SD or range)	Male gender	HTN	CAD	DM	Dys-lipidaemia	Smoking	COPD	CRF	Imaging	CEA	Shunt	CAS	CPD
**SPACE-2** ^12^ 2016 36 centres Austria, Germany, Switzerland	CEA: 70 ± 7 CAS: 69 ± 8 BMT:68 ± 7	CEA: 74% CAS: 73% BMT:77%	CEA: 87% CAS: 90% BMT: 90%	CEA: 34% CAS: 37% BMT: 35%	CEA: 25% CAS: 30% BMT: 35%	CEA: 76% CAS: 80% BMT: 81%	Ever Smoked CEA: 66% CAS: 66% BMT: 80%	No records	No records	Duplex	No records	No records	No records	No records
**ACT 1** ^13^ 2016 65 centres US	CEA: 67.9 ± 6.9 CAS: 67.7 ± 7.0	CEA: 56.9% CAS: 61.2%	CEA: 89.6% CAS: 90.6%	CEA: 51.1% CAS: 53.4%	CEA: 32.4% CAS: 35.6%	CEA: 87.9% CAS: 90.0%	Ever Smoked CEA: 71.2% CAS: 73.7% Current Smoker CEA: 19.5% CAS: 24.4%	CEA: 9.3% CAS: 11.9%	CEA: 6.6% CAS: 8.4%	Duplex and/or DSA	No records	No records	Nitinol stents	All patients
**Mannheim Trial** ^14^ 2016 Haifa-Israel	CEA: 68 ± 8 CAS: 69 ± 7	CEA: 71% CAS: 66%	CEA: 84% CAS: 85%	CEA: 50% CAS: 49%	CEA: 49% CAS: 47%	CEA: 74% CAS: 82%	Undefined CEA: 29% CAS: 22%	No records	CEA: 9% CAS: 9%	Duplex + CTA or MRA	RA	Surgeon preference	Cordis Stent	All patients
**AMTEC trial** ^15^ 2015 3 centres Russia	CEA: 67.0 ± 7.4 BMT: 66.1 ± 6.8	CEA: 65% BMT: 83%	No records -	CEA: 68% BMT: 75%	CEA: 29% BMT: 21%	No records	Current smoker CEA: 68% BMT: 46%	No records	CEA: 0% BMT: 4%	Duplex (NASCET) + CTA or MRA	GA Eversion CEA (most Patients)	No records	n/a	n/a
**CREST** ^16^ 2011 108 centres US 9 centres Canada	CEA: 69.6 ± 8.1 CAS: 69 ± 8.0	CEA: 67.5% CAS: 63.8%	CEA: 87.9% CAS: 88.2%	CEA: 26.5% CAS: 23.5%	CEA: 33.7% CAS: 32.6%	CEA: 91.1% CAS: 89.7%	Current smoker CEA: 22.2% CAS: 26.1%	No records	No records	Duplex, DSA,CTA,MRA	GA: 87.5% Patch: 68.5%	53.60%	RX Acculink stent	96.10%
**ACST-1** ^10^ 2010 126 centres 30 countries	68 (40–91)	65.50%	65%	No records	20%	No records	No records	No records	No records	Duplex (NASCET)	Surgeon preference	Optional	n/a	n/a
**Kentucky trial** ^17^ 2004 Kentucky US	CEA: 69.9(48–84) CAS: 66.6(49–85)	No records	CEA: 97.6% CAS: 81.4%	CEA: 47.6% CAS: 81.4%	CEA: 11.9% CAS: 16.3%	CEA: 19.0% CAS: 20.9%	Undefined CEA: 88.1% CAS: 93.0%	No records	No records	DSA	GA all patient with TCD	No records	Wallstent or Dynalink	Not used
**SAPPHIRE trial** ^18^ 2004 29 centres US	CEA: 72.6 ± 8.9 CAS: 72.5 ± 8.3	CEA: 67.1% CAS: 66.9%	CEA: 85.1% CAS: 85.5%	CEA: 75.5% CAS: 85.8%	CEA: 27.5% CAS: 25.3%	CEA: 76.9% CAS: 78.5%	CEA: 16.4% CAS: 16.9%	CEA: 13.8% CAS: 17.0%	CEA: 7.5% CAS: 6.0%	Duplex/DSA for CAS group	No record	No records	Nitinol stent (Smart or Precise, Cordis)	all patients
**ACAS trial** ^9^ 1995 39 centres US/Canada	Mean age 67	CEA: 66% BMT: 66%	CEA: 64% BMT: 64%	CEA: 69% BMT: 69%	CEA: 25% BMT: 21%	No records	Current Smoker CEA: 28% BMT: 24%	Lung Disease CEA: 6% BMT: 5%	No records	DSA	Surgeon preference	Surgeon preference	n/a	n/a
**Veterans Affairs trail** ^19^ 1993 11 centres US	CEA: 64.1 ± 6.8 BMT: 64.7 ± 6.7	Only male patients	CEA: 63% BMT: 64%	CEA: 30% BMT: 25%	CEA: 30% BMT: 27%	No records	Current Smoker CEA: 52% BMT: 49% Ever Smoked CEA:95% BMT:91%	No records	No records	DSA	GA	Surgeon preference	n/a	n/a
**MACE trial** ^20^ 1992 Mayo clinic US	CEA <55: 2.8% CEA 55–65: 27.8% CEA >65: 69.% BMT <55: 5.7% BMT 55–65: 22.9% BMT >65: 71.4%	CEA: 55.6% BMT: 60%	CEA: 63.9% BMT: 62.9%	CEA: 41.7% BMT: 40%	CEA: 19.4% BMT: 14.3%	CEA: 44.4% BMT: 65.7%	Current Smoker CEA: 25% BMT: 31.4% Ever Smoked CEA:66.7% BMT:74.3%	No records	No records	Duplex/DSA/ocular pneumo-plethys-mography	No records	No records	n/a	n/a

BMT, best medical therapy; CAD, coronary artery disease; CAS, carotid stenting; CEA, carotid endarterectomy; COPD, chronic obstructive pulmonary disease; CPD, cerebral protection device; CRF, chronic renal failure; CTA, computed tomographic angiography; DM, diabetes mellitus; DSA, digital subtraction angiography; GA, general anaesthetic; HTN, hypertension; ICA, internal carotid artery; MI, myocardial infarction; MRA, magnetic resonance angiography; RA, regional anaesthetic; RCT, randomised controlled trial; SD, standard deviation; TCD, transcranial doppler; TIA, transient ischemic attack.

#### CEA vs. CAS vs. BMT

SPACE-2^12^ is the only RCT that was planned as a three-armed trial, namely BMT alone vs. CEA plus BMT vs. CAS plus BMT. This trial was powered to randomize more than 3000 patients over a 5-year period. Because of slow patient recruitment, the three-arm study design was amended in July 2013 to become two parallel randomized studies (BMT alone vs. CEA plus BMT and BMT alone vs. CAS plus BMT). However, again due to slow recruitment, the trial was ceased after enrolment of 513 patients over a 5-year period (CEA plus BMT, n = 203; CAS plus BMT, n = 197; and BMT alone, n = 113). Patients were followed up for up to five years to produce long-term primary efficacy data.

#### CEA vs. BMT

We identified five RCTs^9,10,15,19,20^ reporting comparative outcomes of CEA vs. BMT for asymptomatic carotid disease, which were published between 1992 and 2015. The overall study population comprised of 5349 patients, 2663 in the CEA group and 2686 in the BMT group. Single antiplatelet therapy in the form of aspirin was used in both treatment groups in all but one trial^20^, in which CEA patients received no antiplatelet therapy. BMT as per current guidelines were used in only one trial^15^, the remaining trail did not provide elements of their BMT protocol. In ACST 1^10^ the use of lipid lowering drugs improved significantly during the study period (initially only 7% and at the end of the trail 82%). Follow up ranged between two and ten years. There were no significant differences in baseline demographics and clinical characteristics between the CEA and BMT groups.

#### CEA vs. CAS

We identified five RCTs^13,14,16–18^ reporting comparative outcomes of CEA vs. CAS for asymptomatic carotid disease, which were published between 2008 and 2016. The studies included a total of 3092 patients; 1181 of them underwent CEA and the remaining 1911 underwent CAS. Patients in the CEA group received single antiplatelet therapy with aspirin, whereas those undergoing CAS received dual antiplatelet therapy (aspirin and clopidogrel). Follow up ranged from three to ten years. There were no significant differences in baseline demographics and clinical characteristics between the CEA and BMT groups.

### Risk of bias assessment

**Figure 2:**
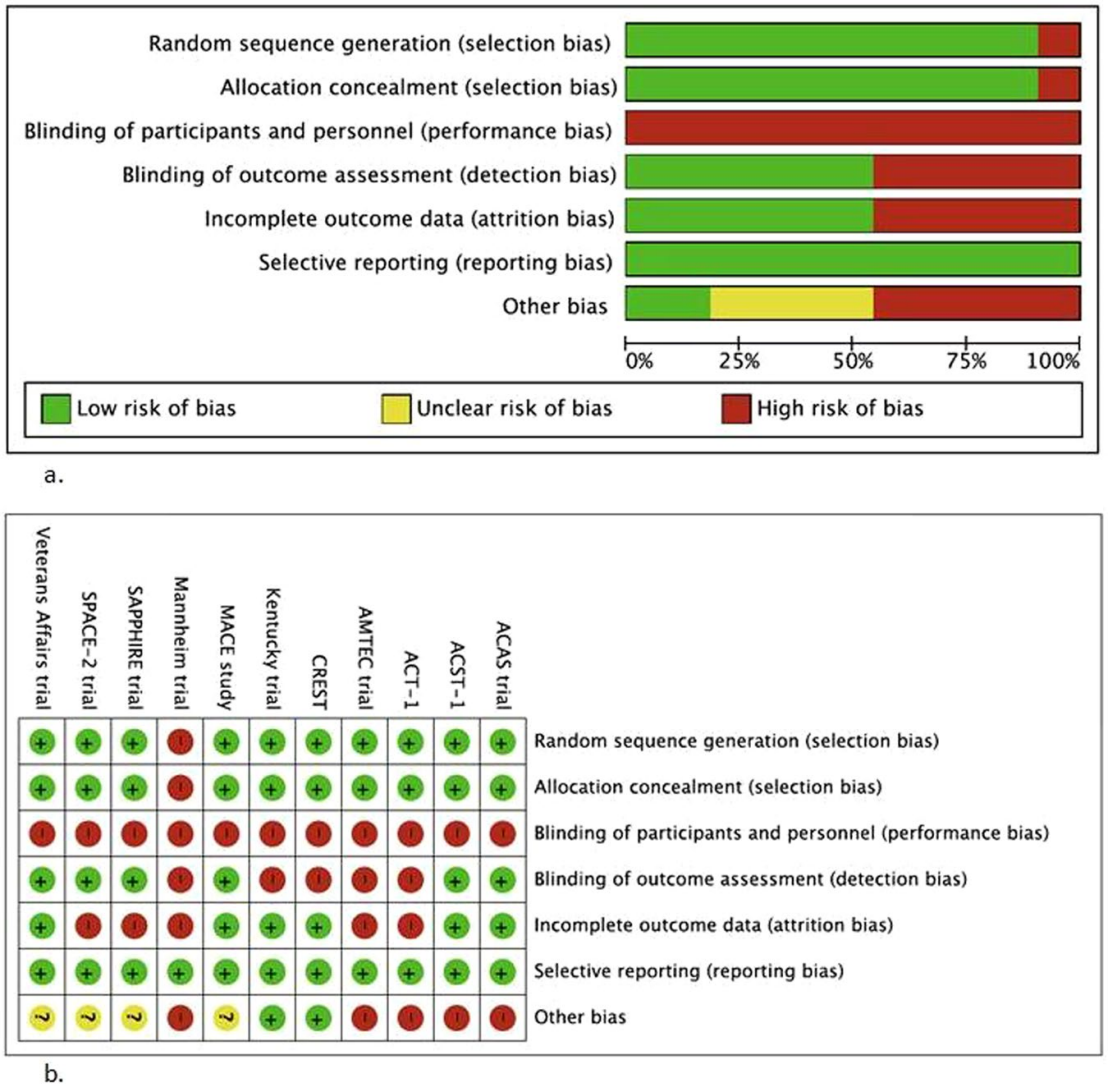
(**a**) Risk of bias graph: review authors’ judgements about each risk of bias item presented as percentages across all included studies. (**b**) Risk of bias summary: review authors’ judgements about each risk of bias item for each included study.

Random sequence generation and allocation concealment methods were adequately described in all but one trial^14^, which did not state patient selection methods. Due to the nature of intervention, blinding of participants and personnel was not possible resulting in a high risk of performance bias. In six RCTs^9,10,12,18–20^, blinding of outcome assessors was adequately described, whereas in the remaining trials, this parameter was inadequately reported. Five RCTs^12,13,15,18,20^ were found to have incomplete outcome data (attrition bias). Four RCTs^12,13,15,18^ were terminated early, three due to slow enrolment and one due to an unacceptable complication rate in the CEA group^20^. No reporting bias was identified in any of the trials. The risk of bias assessment of the RCTs is summarised in Fig. 2.

### Effects of interventions

#### Pair-wise meta-analysis

**Figure 3:**
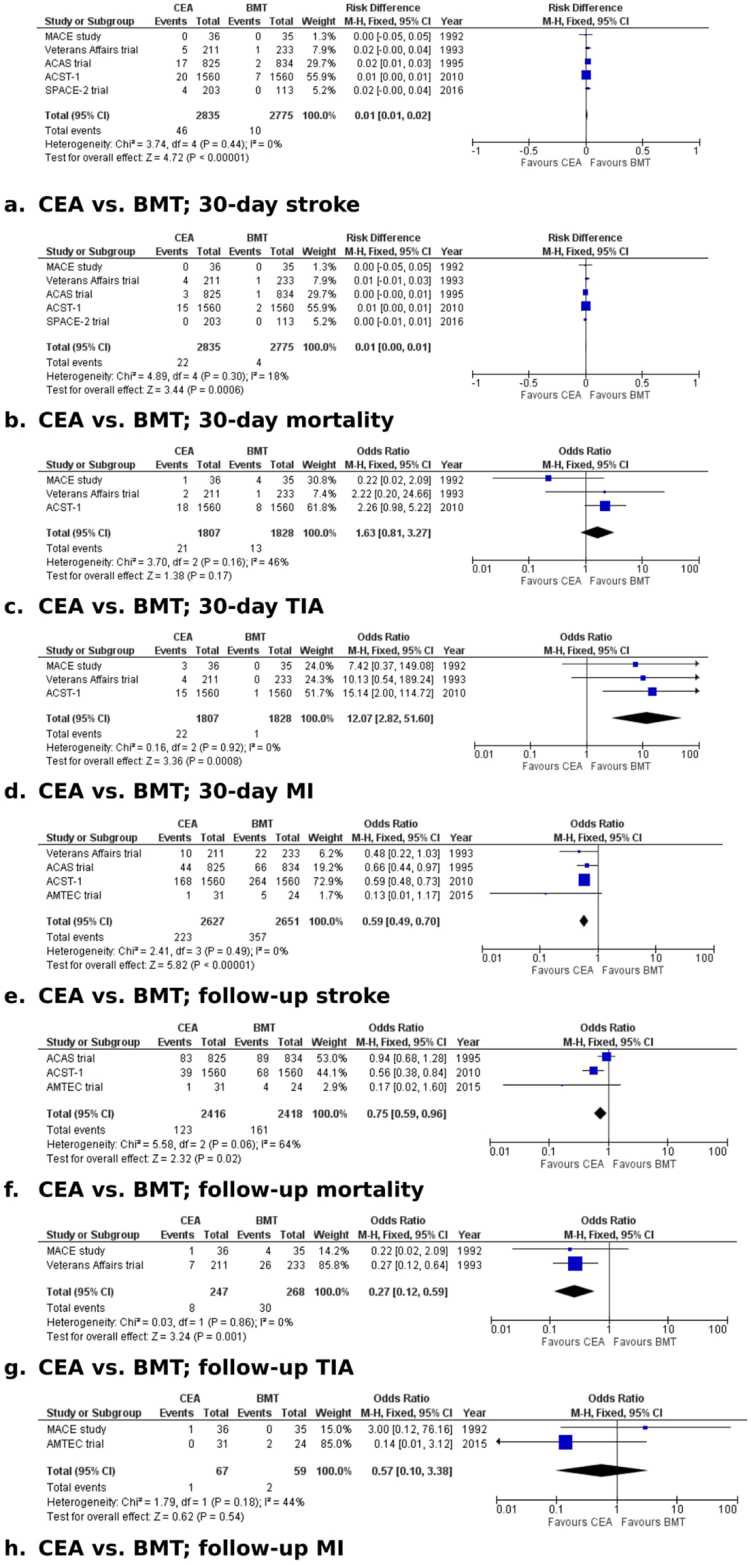
Forest plots of comparisons of CEA vs. BMT). The solid squares denote the odds ratios (ORs) or risk difference (RD). The horizontal lines represent the 95% confidence intervals (CIs), and the diamond denotes the pooled effect size. BMT, best medical therapy; CEA, carotid endarterectomy; M-H, Mantel Haenszel test; MI, myocardial infarction; TIA, transient ischaemic attack.

**Figure 4:**
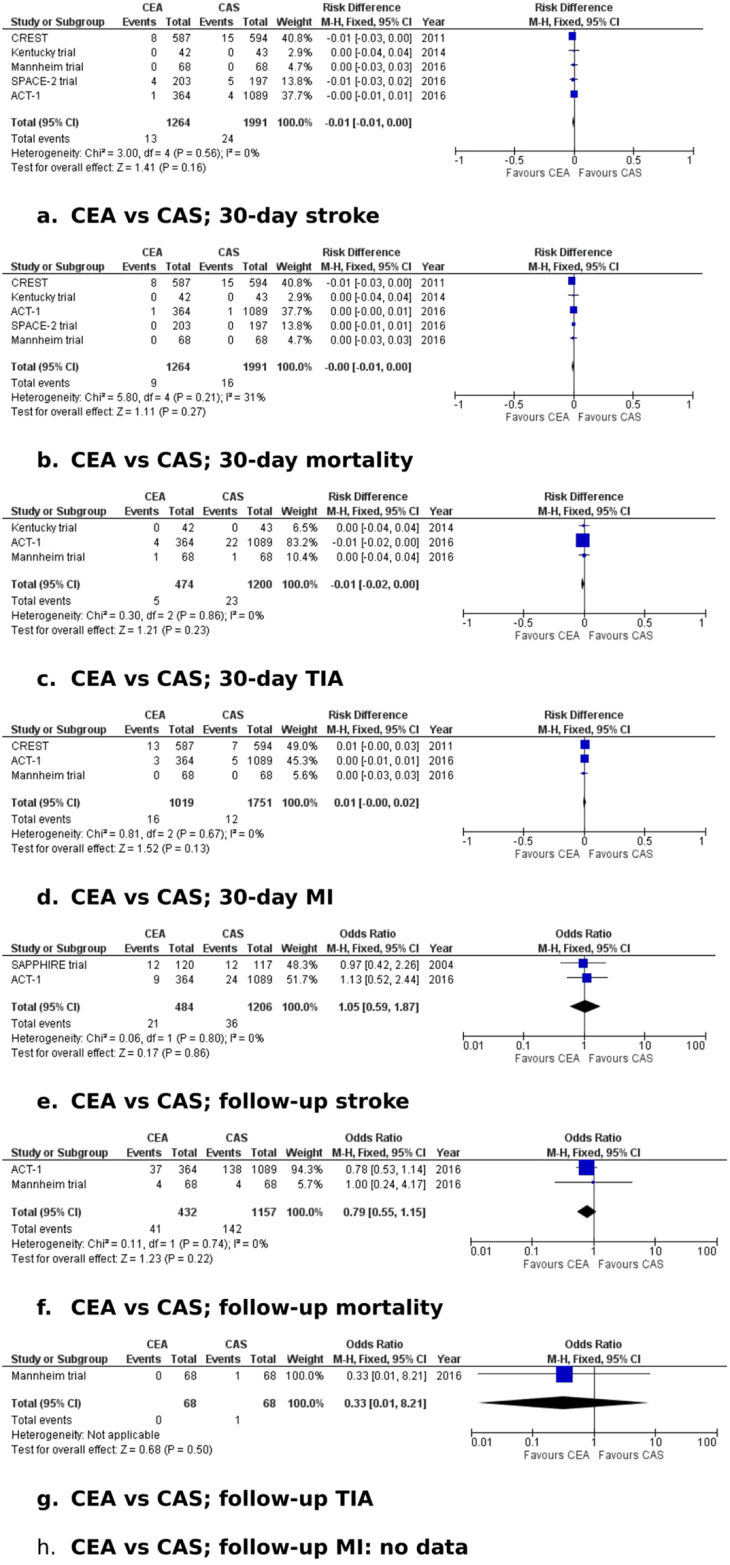
Forest plots of comparisons of CEA vs. CAS. The solid squares denote the odds ratios (ORs) or risk difference (RD). The horizontal lines represent the 95% confidence intervals (CIs), and the diamond denotes the pooled effect size. CAS, carotid stenting; CEA, carotid endarterectomy; M-H, Mantel Haenszel test; MI, myocardial infarction; TIA, transient ischaemic attack.

Forest plots of comparisons of CEA vs. BMT and CEA versus CAS are presented in Figs 3 and 4, respectively.

#### CEA vs. BMT

*Thirty-day ipsilateral stroke*. Data on 30-day ipsilateral stroke were reported in all five studies^9,10,15,19,20^. In the CEA group (2835 patients), the incidence of 30-day ipsilateral stroke was 1.6%, whereas in the BMT group (2775 patients), the incidence was 0.4% (RD: 0.01, 95% CI: 0.01–0.02, P < 0.00001). We found no evidence of heterogeneity among the studies (I^2^ = 0%, P = 0.44). Excluding trials that were judged to be at high risk of bias in two or more domains (ACAS^9^, ACST-1^10^, SPACE-2^12^) showed no difference between the treatment groups (RD: 0.02, 95% CI: −0.00–0.04, P = 0.11). The recruitment period started after 2000 in one trial only (SPACE-2 trial^12^), which found no significant difference in 30-day stroke risk between treatments (OR: 5.12, 95% CI: 0.27–95.97, P = 0.27). The GRADE level of evidence for this outcome was judged to be high.

*Thirty-day mortality*. Data on 30-day mortality were reported in all five studies^9,10,15,19,20^. The 30-day mortality rate was 0.8% in the CEA group (2853 patients) and 0.1% in the BMT group (2775 patients) (RD: 0.01, 95% CI: 0.00–0.01, P = 0.0006). The likelihood of between-study heterogeneity was low (I^2^ = 18%, P = 0.3). Excluding trials that were judged to be at high risk of bias in two or more domains (ACAS^9^, ACST-1^10^, SPACE-2^12^) showed no difference between the treatment groups (RD: 0.01, 95% CI: −0.01–0.03, P = 0.19). The recruitment period started after 2000 in one trial only (SPACE-2 trial^12^), which reported no mortality within 30 days in either group. The GRADE level of evidence for this outcome was judged to be high.

*Thirty-day ipsilateral minor stroke/TIA*. Data on 30-day minor stroke/TIA were reported in three trials^10,19,20^. In the CEA group (1807 patients), the incidence of minor stroke/TIA at 30-days was 1.2%, while in the BMT group (1828 patients), the incidence was 0.7% (OR: 1.63, 95% CI: 0.81–3.27, P = 0.17). There was no evidence of significant statistical heterogeneity among the studies (I^2^ = 46%, P = 0.16). Excluding the trial that was judged to be at high risk of bias in two or more domains (ACST-1^10^) showed no difference between the treatment groups (OR: 0.61, 95% CI: 0.14–2.55, P = 0.5). The recruitment period started before 2000 in all trials included in the analysis. The GRADE level of evidence for this outcome was judged to be high.

*Thirty-day MI*. Data on 30-day MI were reported in three studies^10,19,20^. In the CEA group (1807 patients), MI within 30 days of treatment occurred in 1.2% of patients, whereas in the BMT group (1828 patients), MI within 30 days occurred in 0.1% (OR: 12.07, 95% CI: 2.82–51.6, P = 0.0008). There was no evidence of statistical heterogeneity among the studies (I^2^ = 0%, P = 0.92). Excluding the trial that was judged to be at high risk of bias in two or more domains (ACST-1^10^) showed a difference in favour of BMT (OR: 8.78, 95% CI: 1.08–71.21, P = 0.04). The recruitment period started before 2000 in all trials included in the analysis. The GRADE level of evidence for this outcome was judged to be high.

*Ipsilateral stroke during follow up*. Data on ipsilateral stroke during follow up were reported in four studies^9,10,15,19^. In the CEA group (2627 patients), the incidence of stroke was 8.4%, whereas in the BMT group (2651 patients), ipsilateral stroke occurred in 13.4% of patients (OR: 0.59, 95% CI: 0.49–0.7, P < 0.00001). We found no evidence of statistical heterogeneity among the selected studies (I^2^ = 0%, P = 0.49). Excluding trials that were judged to be at high risk of bias in two or more domains (ACAS^9^, ACST-1^10^, AMTEC^15^) showed no difference between the treatment groups (OR: 0.48, 95% CI: 0.22–1.03, P = 0.06). Only one trial (ACST-1^10^) provided follow-up data >5 years; it found a significance difference in stroke risk in favour of CEA (OR: 0.59, 95% CI: 0.48–0.73, P < 0.00001). The recruitment period started after 2000 in one trial only (AMTEC^15^), which found no significant difference in the follow-up stroke risk between treatments (OR: 0.13, 95% CI: 0.01–1.17, P = 0.07). The GRADE level of evidence for this outcome was judged to be high.

*Mortality during follow up*. Data on long-term mortality were reported in three studies^9,10,15^. The mortality rate during follow up was 5.1% in the CEA group (2416 patients) and 6.6% in the BMT group (2418 patients) (OR: 0.75, 95% CI: 0.59–0.96, P = 0.02). We found no evidence of significant statistical heterogeneity among the studies (I^2^ = 64%, P = 0.06). All three trials included in this analysis were at high risk of bias in two or more domains. Only one trial (ACST-1^10^) provided follow-up data >5 years; it found a significance difference in mortality in favour of CEA (OR: 0.56, 95% CI: 0.38–0.84, P = 0.005). The recruitment period started after 2000 in one trial only (AMTEC^15^), which found no significant difference in mortality risk during follow up between treatments (OR: 0.17, 95% CI: 0.02–1.6, P = 0.12). The GRADE level of evidence for this outcome was judged to be high.

*Ipsilateral minor stroke/TIA during follow up*. Data on long-term ipsilateral minor stroke/TIA were reported in two trials^19,20^. During follow up, ipsilateral minor stroke/TIA occurred in 3.2% in the CEA group (247 patients) and in 1.9% in the BMT group (268 patients) (OR: 0.27, 95% CI, 0.12–0.59, P = 0.001). We identified no evidence of statistical heterogeneity (I^2^ = 0%, P = 0.86). Both trials were found to be of risk of bias in less than two domains. Neither reported follow up >5 years. The recruitment period started before 2000 in both trials included in the analysis. The GRADE level of evidence for this outcome was judged to be high.

*MI during follow up*. Data on MI during follow up were reported in two studies^15,20^. MI during the follow-up period occurred in one patient (1.5%) in the CEA group (67 patients) and in two patients (3.3%) in the BMT group (59 patients) (OR: 0.57, 95% CI: 0.1–3.38, P = 0.54). There was no evidence of statistical heterogeneity between the two studies (I^2^ = 44%, P = 0.18). One trial (AMTEC^15^) was judged to be of high risk of bias in more than two domains; excluding this trial revealed no difference in the risk of MI during follow up between treatment groups (OR: 3, 95% CI: 0.12–76.16, P = 0.51). None of the trials reported follow up >5 years. The recruitment period started after 2000 in one trial only (AMTEC^15^), which found no significant difference in mortality risk during follow up (OR: 0.14, 95% CI: 0.01–3.12, P = 0.22). The GRADE level of evidence for this outcome was judged to be high.

#### CEA vs. CAS

*Thirty-day ipsilateral stroke*. Data on ipsilateral stroke within 30 days of treatment were reported in all five studies^13,14,16–18^. The 30-day incidence of ipsilateral stroke was 1% in the CEA group (1264 patients) and 1.2% in the CAS group (1991 patients) (RD: −0.01, 95% CI: −0.01–0.00, P = 0.16). There was no evidence of statistical heterogeneity among the studies (I^2^ = 0%, P = 0.56). All trials were judged to be at high risk of bias in two or more domains. The recruitment period started before 2000 in one trial (Kentucky);^17^ excluding this trial from the analysis showed no difference in the stroke risk within 30 days between CEA and CAS (RD: −0.01, 95% CI: −0.01–0.00, P = 0.15). The GRADE level of evidence for this outcome was judged to be moderate.

*Thirty-day mortality*. Data on 30-day mortality were reported in all five trials^13,14,16–18^. Mortality was 0.7% in the CEA group (1264 patients) and 0.8% in the CAS group (1991 patients) (RD: −0.00, 95% CI: −0.01–0.00, P = 0.27). We found no evidence of statistical heterogeneity (I^2^ = 31%, P = 0.21). All trials were judged to be at high risk of bias in two or more domains. Excluding the trial where the recruitment period started before 2000 (Kentucky)^17^ revealed no difference in the 30-day mortality risk between CEA and CAS (RD: −0.00, 95% CI: −0.01–0.00, P = 0.26). The GRADE level of evidence for this outcome was judged to be moderate.

*Thirty-day ipsilateral minor stroke/TIA*. Data on ipsilateral minor stroke/TIA occurring within 30 days of treatment were reported in three trials^13,14,17^. In the CEA group (474 patients), the 30-day incidence of minor stroke/TIA was 1.1%, while in the CAS group (1200 patients), it was 1.9% (RD: −0.01, 95% CI: −0.02–0.01, P = 0.23). We found no significant between-study heterogeneity (I^2^ = 0%, P = 0.86). All three trials were judged to be at high risk of bias in two or more domains. Excluding the trial with recruitment starting before 2000 (Kentucky)^17^ revealed no difference in the risk of minor stroke/TIA (RD: −0.01, 95% CI: −0.02–0.00, P = 0.21). The GRADE level of evidence for this outcome was judged to be moderate.

*Thirty-day MI*. Data on MI within 30 days of treatment were reported in three studies^13,14,16^. In the CEA group (1019 patients), MI occurred in 1.6% of patients, whereas in the CAS group (1751 patients), MI within 30 days of treatment occurred in 0.7% (RD: 0.01, 95% CI: −0.00–0.02, P = 0.13). The statistical heterogeneity was insignificant (I^2^ = 0%, P = 0.67). All trials were judged to be at high risk of bias in two or more domains, and their recruitment period started after 2000. The GRADE level of evidence for this outcome was judged to be moderate.

*Ipsilateral stroke during follow up*. Data on long-term ipsilateral stroke were reported in two studies^13,18^. The stroke rate was 4.3% in the CEA group (484 patients) and 3% in the CAS group (1206 patients) (OR: 1.05, 95% CI: 0.59–1.87, P = 0.86). The statistical heterogeneity was low (I^2^ = 0%, P = 0.8). Both trials were judged to be at high risk of bias in two or more domains. None of the trials selected for analysis reported follow up longer than five years. Both trials started recruitment after 2000. The GRADE level of evidence for this outcome was judged to be moderate.

*Mortality during follow up*. Data on long-term mortality were reported in two studies^13,14^. In the CEA group (432 patients), long-term mortality was 9.5%, and in the CAS group (1157 patients), it was 12.3% (OR: 0.79, 95% CI: 0.55–1.15, P = 0.22). There was no significant between-study heterogeneity (I^2^ = 0%, P = 0.74). Both trials were judged to be at high risk of bias in two or more domains. None of the trials selected for analysis reported follow up longer than five years. Both trials started recruitment after 2000. The GRADE level of evidence for this outcome was judged to be moderate.

*Ipsilateral minor stroke/TIA during follow up*. Data on long-term ipsilateral minor stroke/TIA were reported in one trial^14^. In the CEA group (68 patients), no minor ipsilateral stroke/TIA occurred during follow up, and in the CAS group (68 patients), one patient developed a TIA during the follow-up period (1.5%) (OR: 0.33, 95% CI: 0.01–8.21, P = 0.5). The GRADE level of evidence for this outcome was judged to be low.

*MI during follow up*. No data on MI during follow up were reported in the selected trials.

#### CAS vs. BMT

There was no RCT comparing clinical outcomes between CAS and BMT.

#### Network meta-analysis

The geometry of the network of interventions for the primary outcomes yielded an open network without closed loops, as only the SPACE-2^12^ trial included all three treatment groups, reporting 30-day outcome data only. This has led to limited diversity. Excluding the SPACE-2 trial^12^, each comparison is represented only by indirect comparisons (CEA vs BMT or CEA vs CAS); no trial provided direct evidence between CAS and BMT.

All but two studies^15,18^ reported 30-day mortality data. Considering BMT as the reference treatment, ORs and 95% CIs for 30-day mortality were as follows: CAS, 8.41 (2.16–32.71) and CEA, 5.42 (1.85–15.95) with the result favouring BMT (Table 3). For this outcome parameter, the probability of BMT to be the best treatment was 99.9%.

**Table 3:** Network meta-analyses league table.

	BMT	CAS	CEA
30-day mortality			
BMT	*99.9 (99.8%)*	**0.12 (0.03, 0.46)**	**0.18 (0.06, 0.54)**
CAS	**8.41 (2.16, 32.71)**	*7.7 (0.2%)*	1.55 (0.68, 3.54)
CEA	**5.42 (1.85, 15.95)**	0.65 (0.28, 1.47)	*42.4 (0.0%)*
Long-term mortality			
BMT	*20.1(5.8%)*	1.19 (0.54, 2.61)	1.43 (0.89, 2.30)
CAS	0.84 (0.38, 1.84)	*47.6 (27.6%)*	1.20 (0.65, 2.21)
CEA	0.70 (0.43, 1.12)	0.83 (0.45, 1.53)	*82.3 (66.6%)*
30-day ipsilateral stroke			
BMT	*100 (100%)*	**0.15 (0.06, 0.39**)	**0.24 (0.12, 0.48)**
CAS	**6.83 (2.60, 17.97)**	*4.3 (0%)*	1.65 (0.81, 3.34)
CEA	**4.15 (2.09, 8.22)**	0.61 (0.30, 1.23)	*45.7 (0%)*
Long-term ipsilateral stroke			
BMT	*1.8 (0%)*	1.73 (0.95, 3.15)	**1.69 (1.41, 2.02)**
CAS	0.58 (0.32, 1.05)	*75.0 (53.6%)*	0.97 (0.55, 1.72)
CEA	**0.59 (0.50, 0.71)**	1.03 (0.58, 1.82)	*73.2 (46.4%)*
30-day myocardial infarction			
BMT	*79.9 (60.7%)*	0.69 (0.05, 9.94)	**0.09 (0.01, 0.65)**
CAS	1.45 (0.10, 20.77)	*69.0 (39.3%)*	**0.13 (0.02, 0.79)**
CEA	11.04 (1.53, 79.53)	7.64 (1.27, 46.04)	*1.2 (0.0%)*
30-day ipsilateral minor stroke/TIA			
BMT	*87.5 (76.4%)*	**0.42 (0.19, 0.93)**	0.66 (0.21, 2.08)
CAS	**2.37 (1.07, 5.26)**	*9.5 (22.3%)*	1.56 (0.62, 3.97)
CEA	1.52 (0.48, 4.77)	0.64 (0.25, 1.62)	*52.9 (1.3%)*
Long-term ipsilateral minor stroke/TIA			
BMT	*24.3 (0.2%)*	1.08 (0.04, 29.89)	**3.29 (1.45, 7.46)**
CAS	0.93 (0.03, 25.64)	*38.5 (48.1%)*	3.04 (0.01, 8.21)
CEA	**0.30 (0.13, 0.69)**	0.33 (0.01, 8.21)	*87.2 (51.7%)*

Estimates are presented as odds ratio (OR) with 95% confidence interval (CI) in parentheses. ORs above 1 suggest that the treatment listed in the upper row is superior; ORs below 1 suggest that the treatment listed in the left column is superior. Surface under the cumulative ranking curve values (SUCRAs) are given in the diagonal and the probability of being the best treatment in parentheses. Statistically significant values are given in bold. BMT, best medical therapy; CAS, carotid stenting; CEA, carotid endarterectomy; TIA, transient ischaemic attack.

Five trials provided data on long-term mortality^9,10,13–15^. With CEA as the reference, ORs and 95% CIs for long-term mortality were as follows: BMT, 1.43 (0.89–2.30) and CAS, 1.20 (0.65–2.21), with the result favouring CEA without reaching statistical significance (Table 3). For long-term mortality, the probabilities of CEA, CAS and BMT to be the best treatment were 66.6%, 27.6% and 5.8% respectively.

All but one trial^15^ reported data on 30-day ipsilateral stroke. With BMT as reference treatment, ORs and 95% CIs for 30-day ipsilateral stroke were as follows: CAS, 2.37(1.07–5.26) and CEA, 1.52(0.48–4.77), with the result favouring BMT (Table 3). For this outcome, the probability of BMT to be the best treatment was 100%.

Six trials provided data on long-term ipsilateral stroke^9,10,13,15,18,19^. With CEA as the reference, ORs and 95% CIs for long-term ipsilateral stroke were as follows: BMT, 1.69 (1.41–2.02) and CAS, 0.97 (0.55–1.72), with statistically significant results in favour of CEA over BMT only (Table 3). For ipsilateral stroke during follow up, the probabilities of CEA, CAS and BMT to be the best treatment were 46.4%, 53.6% and 0%, respectively.

The results from network meta-analysis predictive interval showed that future trails are highly likely to change the direction of the treatment effect for patients with asymptomatic carotid disease, particularly so for BMT.

## Discussion

We conducted a pairwise and network meta-analysis of treatment strategies for asymptomatic carotid disease including a total of 8954 patients from 11 randomised clinical trials. Pairwise treatment meta-analysis showed that CEA is superior to BMT in reducing the risk of long-term mortality and stroke. There were no statistical significant differences between CEA and CAS in terms of peri-interventional or long term ipsilateral stoke/mortality.

The value of CEA in reducing the risk of stroke in asymptomatic carotid artery stenosis is predominantly based on two seminal studies performed in the 1990s. The ACAS^9^ was a well-conducted RCT that was halted at 2.7 years because of a projected 5.9% absolute risk reduction at five years favouring CEA. BMT in this trail were only in the form of Aspirin and a general discussion in cardiovascular risk reduction factors. The ACST trial^10^ randomized 3120 asymptomatic carotid artery stenosis patients to immediate CEA or delayed surgery for symptoms only. Combining perioperative events and strokes, net risks were 6.9% vs. 10.9% at 5 years and 13.4% vs. 17.9% at 10 years.BMT improved significantly during these ten years with 82% of patient on lipid lowering by drugs by the end of trail compare to only 7% at the beginning of the trail.

In 2016, the results from two large RCTs comparing CEA with CAS in asymptomatic carotid stenosis patient were published. The CREST trial^21^ and ACT 1^13^ resolved the durability dilemma with CAS. However, these trials did not resolve the issue of generalisability of these findings into routine clinical practice, where rates of death and stroke may be much higher among patients undergoing carotid stenting^22^.

In CEA vs. BMT, the quality of the evidence was judged to be high according to the GRADE system. This was reduced to moderate/low for CEA vs. CAS as all trials included in the analysis were judged to be at high risk of bias in two or more domains.

Systematic review^23^ of 47 studies (6 RCTs and 41 observational studies) investigating the evidence on management strategies for asymptomatic carotid stenosis also concluded that the evidence was not sufficiently robust or applicable to current clinical practice to allow clinicians to draw confident conclusions on the comparative effectiveness of management strategies for adults with asymptomatic carotid stenosis. Moresoli *et al*.^24^, published a meta-analysis of 11 studies (5 RCTs and 6 observational studies) on CAS vs CEA in asymptomatic carotid disease. The results of this meta-analysis corroborate our findings as there was no clinically significant differences between treatments for long-term stroke (RR, 1.24; 95% CI, 0.76–2.03) and the composite outcome of periprocedural stroke, death or MI, or long-term ipsilateral stroke (RR, 0.92; 95% CI, 0.70–1.21). Most recently, Kakkos SK *et al*.^25^ published a meta-analysis of 9 RCTs comparing CEA with CAS in asymptomatic carotid disease patients. Regarding the long-term outcome of stroke or death rate at 30 days plus ipsilateral stroke during follow-up, this was significantly higher for CAS (3.64%) than for CEA (2.45%) (OR, 1.51; 95% CI, 1.02–2.24; P = 0.04; I2 = 0%). However, quality of evidence for all stroke outcomes was graded moderate.

The network meta-analysis is the first to be undertaken in this area. It demonstrates that CAS and CEA increase the risk of death and ipsilateral stroke at 30 days compared to BMT. This is an unsurprising finding given the well reported risks of revascularisation interventions. The network meta-analysis results favour CEA in the long term, but this finding is less conclusive.

CREST-2^26^ and ACST-2^27^ trials are currently running to provide level 1 evidence in regards of treatment strategy for asymptomatic carotid artery disease. CREST-2 consists of 2 parallel, RCTs. One trial will compare BMT to CEA BMT. The parallel trial will compare BMT to CAS plus BMT. An estimated 2480 participants will be enrolled in CREST-2 at approximately 120 sites in the United States and in several Canadian sites. ACST-2 is a large international RCT comparing CEA versus CAS in patients with asymptomatic carotid stenosis. ACST-2 is currently recruiting patients from over 112 centres in over 20 countries worldwide. The trial is on track to recruit 3600 patients by 2019.

The results of our review should be interpreted with caution in view of the following limitations. The two major limitations to the pair-wise meta-analysis are variation in follow up period together with significant heterogeneity in BMT within and between the studies. Follow up period were between 30-days and 9 years. This variation will have an effect in reporting outcome measures using OR. This is particularly true when event rate is low, as in carotid trials. In terms of BMT, only one trail^15^ adhered to current guidelines of aggressive medical therapy (blood pressure control, DM treatment, lipid lowering agents) together with lifestyle modification (exercise, smoking cessation and weight reduction). Antiplatelet were the only components of BMT in earlier trails with introduction to statin treatment in late 90s. No sufficient data are provided by the randomised clinical trials included in our review to allow us to perform meta-regression analysis to investigate the effect of statin and/or aspirin on the outcomes. In addition, within CEA/CAS trial patients received different BMT regime (dual antiplatelet treatment if were allocated to CAS). However we tried to overcome these limitations by conducting sensitivity analysis excluding old studies and those with <5 years follow up.

There were also limitations in the NMA. The network geometry did not provide any closed loops across the competing interventions except for one trial that provided data on 30-day ipsilateral stroke only; therefore, we were not able to assess inconsistency between direct and indirect evidence. There was no direct comparison between CAS and BMT. There was a different time span between trials comparing CEA vs. BMT (1997–2015) and CEA vs. CAS (2004–2016), which might challenge transitivity (assumption of similarity in the study characteristics between trails). Transitivity might be also challenged by the variation in the components of BMT and variation in the inclusion criteria. Similarly, the difference in follow up across trials and across the network may introduce heterogeneity and inconsistency, respectively.

## Conclusions

Surgical intervention with CEA is superior to BMT in preventing long term ipsilateral stroke/mortality in asymptomatic carotid disease, but there is probably no difference between CEA and CAS. CREST-2 will clarify whether revascularization interventions provide long-term benefit to patients treated by current best-available medical therapy.

## Methods

### Design

This systematic review followed the guidelines of the Preferred Reporting Items for Systematic reviews and Meta-Analyses (PRISMA) Extension Statement for Reporting of Systematic Reviews Incorporating Network Meta-analyses of Health Care Interventions^28^. The International Prospective Register of Systematic Reviews (PROSPERO) registration number of this protocol is CRD 42016046153.

### Criteria for considering studies for this review

#### Types of studies

Only randomised controlled trials (RCTs) investigating the outcomes of CEA, CAS and BMT in asymptomatic carotid disease were considered in this review.

#### Types of participants

We included any patients (no age or gender restriction) diagnosed with carotid stenosis >50% without any neurological symptoms indicating a cerebrovascular event during the 180 days preceding initiation of treatment for carotid disease. The diagnosis of carotid disease should have been established with objective diagnostic measures, e.g. duplex ultrasonography, magnetic resonance (MR) angiography, digital subtraction angiography (DSA), or computed tomographic (CT) angiography.

#### Types of interventions

We planned to compare outcomes of patients with asymptomatic carotid disease undergoing treatment with CEA, CAS and BMT. CEA could have been performed under general or local anaesthesia. Any technique of CEA was considered including conventional endarterectomy with direct or patch closure or the eversion technique. CAS could have been performed with or without a cerebral protective device (CPD). We considered any type of stent including a closed or open cell design. BMT was implemented according to evidence-based guidelines; it mainly consisted of optimal antiplatelet therapy according to clinical practice at the participating centres, cholesterol-lowering agents (e.g. statin), antihypertensive medication and targeted risk factor modification.

#### Types of outcome measures

*Primary outcomes*

- Death and stroke occurring within 30 days of treatment or during the hospital stay for the index procedure (CEA or CAS) and during follow up.
*Secondary outcomes*
- Myocardial infarction (MI) occurring in the perioperative period (within 30 days or during the hospital stay) and during follow up.
- Transient ischemic attack (TIA) occurring in the perioperative period (within 30 days or during the hospital stay) and during follow up.

### Search methods for identification of studies

The literature search strategy was developed in consultation with a clinical information specialist. Eligible studies were identified by searching Cochrane Central Register of Controlled Trials (CENTRAL), Excerpta Medica database (EMBASE), U.S. National Library of Medicine’s database (MEDLINE), Cumulative Index to Nursing and Allied Health Literature (CINAHL). World Health Organization (WHO) International Clinical Trials Registry and the ISRCTN Register was searched for on-going clinical trials. The final search was carried out in October 2016. The search was not restricted to any language. The literature search strategy is presented in Appendix I.

### Data extraction and management

Two review authors (MB, IR) independently evaluated the studies and selected the studied that fulfilled the inclusion criteria for this review. A third author (GAA) then assess all the selected studie and ensure their eligibility for the inclusion criteria and also acted as an adjudicator in the event of disagreement.

We developed a data extraction sheet, which was pilot-tested and refined accordingly. One review author (MB) extracted the data from the selected studies and a second review author (IR) crosschecked the collected data. Disagreements were resolved by discussion between the authors. The following data were collected:

- Study-related information (first author, year of publication, single-centre or multi-centre study).
- Baseline demographics and clinical characteristics of the study populations (age, gender, diabetes mellitus, hypertension, type of carotid intervention, type of antiplatelet therapy).
- Outcome data, as outlined above.

### Assessment of risk of bias in included studies

We applied the Cochrane tool for the assessment of the risk of bias of the selected trials^29^. Briefly, this tool evaluates six main domains: random sequence generation and allocation concealment (selection bias), blinding of participants and personnel (performance bias), blinding of outcome assessment (detection bias), incomplete outcome data (attrition bias), selective reporting (reporting bias), and other sources of bias. For each individual domain, we classified studies into low, unclear, or high risk of bias.

The Grades of Recommendation, Assessment, Development and Evaluation (GRADE) working group system was utilised for grading the quality of evidence as high, moderate, low and very low, based on directness of evidence, within-study risk of bias, precision of effects estimates, heterogeneity, and risk of publication bias^30^.

### Methods of analysis

We used the RevMan 5.3 software (Cochrane collaboration, Copenhagen, Denmark) for pair-wise meta-analyses. Treatment effect estimates were calculated using the odds ratio (OR) or risk difference (RD) and 95% confidence interval (CI) to reflect the uncertainty of point estimate of effects. We based calculations using an intention-to-treat approach and all randomized participants were included in the analysis regardless of loss to follow up. The unit of analysis was the individual patient. For data synthesis, we used a fixed-effect model to calculate the pooled treatment effect and 95% CI for dichotomous outcome variables. We used a random-effects model when we found significant heterogeneity (defined as I^2^ greater than 75%). We created a forest plot for each treatment effect.

We assessed inter-study heterogeneity visually using a forest plot. We also calculated the I^2^ statistic to measure the amount of interstudy heterogeneity. We considered I^2^ values less than 50% as indicative of low heterogeneity, I^2^ values between 50% and 75% as indicative of moderate heterogeneity, and I^2^ values greater than 75% as indicative of significant heterogeneity.

We planned to construct a funnel plot to test for reporting bias in meta-analyses that included 10 or more studies. We planned to assess publication bias visually evaluating the symmetry of the funnel plots. We also planned to mathematically estimate publication bias using the Egger’s regression intercept.

We performed sensitivity analysis by sequentially excluding trials with a high risk of bias in two or more domains and performed a pooled sensitivity analysis in order to assess whether the included studies, deemed to be biased, impacted the final analysis. We also performed separate analysis of follow-up outcome data for studies providing follow up longer than five years. We performed sensitivity analysis excluding old studies (where the recruitment period started before 2000).

We performed a network meta-analysis in Stata version 13 (College Station, Texas, USA) using the *network* command and self-programmed Stata routines^31–33^. We used the restricted maximum likelihood method to estimate heterogeneity assuming a common estimate for the heterogeneity variance across the different comparisons. We estimated the ranking probabilities for all treatments of being at each possible rank for each intervention. We obtained a hierarchy of the competing interventions using rankograms and the surface under the cumulative ranking curve (SUCRA) and mean ranks^34^. We produced the relevant plots using the suite of Stata commands by Chaimani *et al*.^33^.

### Data availability

The data spread sheet generated during and/or analysed during the current study are available from the corresponding author on reasonable request.

## Electronic supplementary material

Supplementary Information

## References

[1] The Top Ten Causes of Death- Fact Sheet No. 310. Geneva, World Health Organization (2011).

[2] British Geriatrics Society. Human and economic burden of stroke. Age and Ageing. **38**, 4–5 (2009). 10.1093/ageing/afn282 19141505

[3] Petty GW (1999). Ischemic stroke subtypes: a population-based study of incidence and risk factors. Stroke..

[4] Barnett HJ (2000). Causes and severity of ischemic stroke in patients with internal carotid artery stenosis. JAMA..

[5] The Asymptomatic Carotid Surgery Trial (ACST) Collaborative Group. 10-year stroke prevention after successful carotid endarterectomy for asymptomatic stenosis (ACST-1): a multicentre randomised trial. *Lancet*. **376**, 1074–84 (2010). 10.1016/S0140-6736(10)61197-X PMC2956884 20870099

[6] Zhang L (2015). Systematic Review and Meta-Analysis of Carotid Artery Stenting Versus Endarterectomy for Carotid Stenosis: A Chronological and Worldwide Study. Medicine (Baltimore)..

[7] Ricotta JJ (2011). Updated Society for Vascular Surgery guidelines for management of extracranial carotid disease. J Vasc Surg..

[8] Goldstein LB (2011). Guidelines for primary prevention of stroke. Guideline for healthcare professionals from AHA/ASA. Stroke..

[9] Executive Committee for the Asymptomatic Carotid Atherosclerosis Study. Endarterectomy for asymptomatic carotid artery stenosis. *JAMA*. **273**, 1421–8 (1995). 7723155

[10] Asymptomatic Carotid Surgery Trial Collaborators. The MRC Asymptomatic Carotid Surgery Trial (ACST): carotid endarterectomy prevents disabling and fatal carotid territory strokes. *Lancet*. **363**, 1491–502 (2004).

[11] Marquardt L, Geraghty OC, Mehta Z, Rothwell PM (2010). Low risk of ipsilateral stroke in patients with asymptomatic carotid stenosis on best medical treatment: a prospective, population-based study. Stroke..

[12] Eckstein HH (2016). SPACE 2 Investigators. SPACE-2: A Missed Opportunity to Compare Carotid Endarterectomy, Carotid Stenting, and Best Medical Treatment in Patients with Asymptomatic Carotid Stenoses. Eur J Vasc Endovasc Surg..

[13] ACT I Investigators. Randomized Trial of Stent versus Surgery for Asymptomatic Carotid Stenosis. *N Engl J Med***. 374**, 1011–20 (2016). 10.1056/NEJMoa1515706 26886419

[14] Mannheim D, Karmeli R. A prospective randomized trial comparing endarterectomy to stenting in severe asymptomatic carotid stenosis. *J Cardiovasc Surg*. [Epub ahead of print] (2016). 10.23736/S0021-9509.16.09513-6 27332677

[15] Asymptomatic Carotid Artery Stenosis (AMTEC) Study Group. Modern medical treatment with or without carotid endarterectomy for severe asymptomatic carotid atherosclerosis. *J Vasc Surg***. 62**, 914–22 (2015). 10.1016/j.jvs.2015.05.005 26410046

[16] CREST Investigators (2011). Safety of stenting and endarterectomy by symptomatic status in the Carotid Revascularization Endarterectomy Versus Stenting Trial (CREST). Stroke..

[17] Brooks WH, McClure RR, Jones MR, Coleman TL, Breathitt L (2004). Carotid angioplasty and stenting versus carotid endarterectomy for treatment of asymptomatic carotid stenosis: a randomized trial in a community hospital. Neurosurgery..

[18] SAPPHIRE Investigators (2008). Long-term results of carotid stenting versus endarterectomy in high-risk patients. N Engl J Med..

[19] Hobson RW (1993). Efficacy of carotid endarterectomy for asymptomatic carotid stenosis. The Veterans Affairs Cooperative Study Group. N Engl J Med..

[20] Mayo Asymptomatic Carotid Endarterectomy Study Group. Results of a randomized controlled trial of carotid endarterectomy for asymptomatic carotid stenosis. *Mayo Clin Proc*. **67**, 513–8 (1992). 10.1016/s0025-6196(12)60456-x 1434877

[21] CREST investigators. Long-Term Results of Stenting versus Endarterectomy for Carotid-Artery Stenosis. *N Engl J Med*. **374**, 1021–31 (2016). 10.1056/NEJMoa1505215 PMC4874663 26890472

[22] Spence JD, Naylor AR (2016). Endarterectomy, Stenting, or Neither for Asymptomatic Carotid-Artery Stenosis. N Engl J Med..

[23] Raman G (2013). Management strategies for asymptomatic carotid stenosis: a systematic review and meta-analysis. Ann Intern Med..

[24] Moresoli P. *et al*. Carotid Stenting Versus Endarterectomy for Asymptomatic Carotid Artery Stenosis: A Systematic Review and Meta-Analysis. *Strok*e. [Epub ahead of print]. 10.1161/STROKEAHA.117.016824 (2017). 10.1161/STROKEAHA.117.016824 28679848

[25] Kakkos SK, Kakisis I, Tsolakis IA, Geroulakos G (2017). Endarterectomy achieves lower stroke and death rates compared with stenting in patients with asymptomatic carotid stenosis. J Vasc Surg..

[26] CREST-2 trial. 2014. Available from: http:// http://www.crest2trial.org. Last accessed September (2017).

[27] ACST-2 trail. 2007. Available from: https://acst-2.org. Last accessed September (2017).

[28] Liberati A (2009). The PRISMA statement for reporting systematic reviews and meta-analyses of studies that evaluate healthcare interventions: explanation and elaboration. BMJ..

[29] Higgins, J. P. T. & Green, S. (editors). Cochrane Handbook for Systematic Reviews of Interventions Version 5.1 [updated March 2011]. The Cochrane Collaboration, 2011. www.cochrane-handbook.org (2017).

[30] Guyatt GH (2008). GRADE Working Group. GRADE: an emerging consensus on rating quality of evidence and strength of recommendations. BMJ..

[31] White IR (2015). Network meta- analysis. Stata J..

[32] Chaimani A, Salanti G (2015). Visualizing assumptions and results in network meta-analysis: The network graphs package. Stata J..

[33] Chaimani A, Higgins JP, Mavridis D, Spyridonos P, Salanti G (2013). Graphical tools for network meta-analysis in STATA. PLoS One..

[34] Salanti G, Ades AE, Ioannidis JP (2011). Graphical methods and numerical summaries for presenting results from multiple-treatment meta-analysis: an overview and tutorial. J Clin Epidemiol..

