# Supplementary material for: Systematic review and network meta-analysis of treatment strategies for asymptomatic carotid disease
Source: Sci Rep. 2018 Mar 13;8:4458. doi: 10.1038/s41598-018-22356-z (PMC5849752; doi:10.1038/s41598-018-22356-z)
Supplement: Supplementary file 1 — Supplementary Information [file 41598_2018_22356_MOESM1_ESM.pdf]

Systematic review and network meta-analysis of treatment strategies for asymptomatic carotid disease

[*1,2]Mohamed Barkat MRCS

[1,2]Iain Roy MRCS

[3]Stavros A. Antoniou MD, PhD, FEBS

[1,4]Francesco Torella MD, FRCS

[5]George A. Antoniou MD, PhD, MSc, FEBVS

[1]Liverpool Vascular and Endovascular Service, Royal Liverpool University Hospital, Liverpool, UK

[2]Institute of Ageing and Chronic disease, University of Liverpool, Liverpool, UK

[3]Department of Surgery, University Hospital of Heraklion, University of Crete, Heraklion, Greece

[4]School of Physical Sciences, University of Liverpool, Liverpool, UK

[5]Department of Vascular and Endovascular Surgery, The Royal Oldham Hospital, Pennine Acute Hospitals NHS Trust, Manchester, UK

***corresponding author**

Link 8C, Royal Liverpool University Hospital, Prescot street, Liverpool, L7 8XP

E-mail: mohamed.barkat@liverpool.ac.uk

Appendix I. Search strategy

1. Medline; ASYMPTOMATIC DISEASES/; 2996 results.
2. Medline; asymptomatic.ti,ab; 121257 results.
3. Medline; 1 OR 2; 122279 results.
4. Medline; CAROTID STENOSIS/; 13222 results.
5. Medline; CAROTID ARTERY DISEASES/; 19825 results.
6. Medline; (carotid ADJ (arter* OR stenosis)).ti,ab; 60594 results.
7. Medline; 4 OR 5 OR 6; 73227 results.
8. Medline; 3 AND 7; 5375 results.
9. Medline; ENDARTERECTOMY/ OR ENDARTERECTOMY, CAROTID/; 13830 results.
10. Medline; "carotid endarterectom*".ti,ab; 9032 results.
11. Medline; 9 OR 10; 16090 results.
12. Medline; STENTS/; 54350 results.
13. Medline; ("carotid stent*" OR "carotid artery stent*").ti,ab; 3146 results.
14. Medline; ANGIOPLASTY/; 6203 results.
15. Medline; "carotid angioplast*".ti,ab; 889 results.
16. Medline; 12 OR 13 OR 14 OR 15; 58476 results.
17. Medline; ("conservative treatment*" OR "best medical therap*" OR "medical tratment*" OR "no surgery").ti,ab;
26301 results.
18. Medline; 11 OR 16 OR 17; 97836 results.
19. Medline; 8 AND 18; 2804 results.
20. Medline; 19 [Limit to: (Document type Randomized Controlled Trial)]; 123 results.

1. CINAHL; asymptomatic.ti,ab; 10757 results.
2. CINAHL; CAROTID STENOSIS/; 1630 results.
3. CINAHL; CAROTID ARTERY DISEASES/; 1569 results.
4. CINAHL; (carotid ADJ (arter* OR stenosis)).ti,ab; 4424 results.
5. CINAHL; 2 OR 3 OR 4; 5781 results.
6. CINAHL; 1 AND 5; 600 results.
7. CINAHL; ENDARTERECTOMY/ OR ENDARTERECTOMY, CAROTID/; 1358 results.
8. CINAHL; "carotid endarterectom*".ti,ab; 881 results.
9. CINAHL; CAROTID STENOSIS SURGERY/; 596 results.
10. CINAHL; 7 OR 8 OR 9; 1762 results.
11. CINAHL; STENTS/; 7273 results.
12. CINAHL; ("carotid stent*" OR "carotid artery stent*").ti,ab; 485 results.
13. CINAHL; ANGIOPLASTY/; 1208 results.
14. CINAHL; "carotid angioplast*".ti,ab; 98 results.
15. CINAHL; 11 OR 12 OR 13 OR 14; 8159 results.
16. CINAHL; ("conservative treatment*" OR "best medical therap*" OR "medical treatment*" OR "no surgery").ti,ab;
6604 results.
17. CINAHL; 10 OR 15 OR 16; 15853 results.
18. CINAHL; 6 AND 17; 328 results.
19. CINAHL; RANDOMIZED CONTROLLED TRIALS/; 27761 results.
20. CINAHL; 18 AND 19; 6 results

1. EMBASE; ASYMPTOMATIC DISEASE/; 7431 results.

2. EMBASE; asymptomatic.ti,ab; 165045 results.
3. EMBASE; 1 OR 2; 167752 results.
4. EMBASE; CAROTID ARTERY OBSTRUCTION/; 22168 results.
5. EMBASE; CAROTID ARTERY DISEASE/; 10567 results.
6. EMBASE; (carotid ADJ (arter* OR stenosis)).ti,ab; 79476 results.
7. EMBASE; 4 OR 5 OR 6; 90547 results.
8. EMBASE; 3 AND 7; 7146 results.
9. EMBASE; ENDARTERECTOMY/ OR CAROTID ENDARTERECTOMY/; 22164 results.
10. EMBASE; "carotid endarterectom*".ti,ab; 11898 results.
11. EMBASE; CAROTID ARTERY SURGERY/; 2038 results.
12. EMBASE; 9 OR 10 OR 11; 24625 results.
13. EMBASE; CAROTID ARTERY STENT/ OR CAROTID ARTERY STENTING/; 4685 results.
14. EMBASE; ("carotid stent*" OR "carotid artery stent*").ti,ab; 5058 results.
15. EMBASE; CAROTID ANGIOPLASTY/; 494 results.
16. EMBASE; "carotid angioplast*".ti,ab; 1266 results.
17. EMBASE; 13 OR 14 OR 15 OR 16; 7519 results.
18. EMBASE; ("conservative treatment*" OR "best medical therap*" OR "medical treatment*" OR "no surgery").ti,ab;
89669 results.
19. EMBASE; 12 OR 17 OR 18; 117396 results.
20. EMBASE; 8 AND 19; 3855 results.
21. EMBASE; 20 [Limit to: (Clinical Trials Randomized Controlled Trial)]; 181 results.
